# Crosslinked and Dyed Chitosan Fiber Presenting Enhanced Acid Resistance and Bioactivities

**DOI:** 10.3390/polym8040119

**Published:** 2016-04-01

**Authors:** Xiao-Qiong Li, Ren-Cheng Tang

**Affiliations:** 1 College of Textile and Clothing Engineering, Soochow University, 199 Renai Road, Suzhou 215123, China; lixiaoqiongsuda@outlook.com; 2 National Engineering Laboratory for Modern Silk, College of Textile and Clothing Engineering, Soochow University, 199 Renai Road, Suzhou 215123, China

**Keywords:** chitosan fiber, acid resistance, antioxidant activity, antibacterial activity, crosslinking, aziridine crosslinker, lac dye

## Abstract

The application of biodegradable chitosan fiber for healthy and hygienic textiles is limited due to its poor acid resistance in wet processing and poor antioxidant activity. In order to prepare chitosan fiber with good acid resistance and high antioxidant activity, chitosan fiber was first crosslinked by a water-soluble aziridine crosslinker, and then dyed with natural lac dye consisting of polyphenolic anthraquinone compounds. The main application conditions and crosslinking mechanism of the aziridine crosslinker, the adsorption mechanism and building-up property of lac dye on the crosslinked fiber, and the effects of crosslinking and dyeing on the antioxidant and antibacterial activities of chitosan fiber were studied. The crosslinked fiber exhibited greatly reduced weight loss in acidic solution, and possessed excellent acid resistance. Lac dye displayed a very high adsorption capability on the crosslinked fiber and a high utilization rate under weakly acidic medium. The Langmuir–Nernst isotherm was the best model to describe the adsorption behavior of lac dye, and Langmuir adsorption had great contribution to total adsorption. Lac dyeing imparted good antioxidant activity to chitosan fiber. Crosslinking and dyeing had no impact on the good inherent antibacterial activity of chitosan fiber.

## 1. Introduction

In recent years, chitosan as a biological polymer has drawn much attention because of its unique properties such as nontoxicity, biocompatibility, biodegradability and antibacterial activity [1,2], and found its bioapplications in a large variety of important fields, such as drug delivery, tissue engineering, artificial skin, ocular bandage lenses, antibacterial agents, wound dressing and other biomedical aspects [1,2,3]. Chitosan can be used to manufacture a fibrous product by wet spinning [1,4]. The fibers made of chitosan are useful as absorbable sutures and wound dressing materials. Nowadays, chitosan fiber has become one of the most important biobased and functional textile fibers. Chitosan fiber for the purpose of textile applications has some inherent advantages such as antibacterial activity, deodorizing ability, organism compatibility, wound-healing, moisture-retaining, and gentleness to the body [4], and it has been applied to develop functional textiles including underwear, sleepwear, sportswear, socks, towel, tablecloth, upholstery, medical clothing, wound-dressings, and cosmetic films [4,5]. In most cases, chitosan fiber is blended with other staple fibers for the development of functional products in textile industry.

The use of chitosan materials in some biomedical fields and textile industry has been restricted due to their inherent water susceptibility and relatively low stiffness and strength, especially in moist environments and acidic media [2,6,7,8]. Chemical crosslinking modification has been the most common approach to improve the wet stability of chitosan [6,7]. Various types of crosslinking modifications are used depending on the end-uses of chitosan materials and the requirements of improvement in properties desired. The reagents used in crosslinking treatments involve traditional dialdehydes, epoxy compounds, 1-ethyl-3-(3-dimethylaminopropyl)-carbodiimide (EDAC)/*N*-hydroxyl succinimide (NHS), *etc.* [9,10,11]. Apart from the traditional crosslinking treatments, more recently, some nanocomposite chitosan scaffolds were found to possess increased stability in wet states. For instance, chitosan–graphene oxide network structure scaffolds, which were synthesized by covalent linkage of the carboxyl groups in graphene oxide with the amine groups in chitosan in virtue of the action of EDAC/NHS, had good shape retention ability in acidic and alkaline solutions and good stability against enzymatic degradation [12]. Using a similar approach, chitosan–single walled carbon nanohorn hybrid scaffolds with enhanced stability under aqueous environments were able to be prepared in the presence of EDAC/NHS [13,14]. Additionally, chitosan/nanohydroxyapatite hybrid nanocomposite scaffolds, which were prepared by grafting of chitosan with propylene oxide, and then linking with ethylene glycol functionalized nanohydroxyapatite, also showed good shape retention ability and stability in solutions of different pHs including 7.4, 1.2 and 14 [15]. Although the good stability of chitosan in wet states can be imparted by chemical modifications, studies have shown that the synthetic crosslinking reagents are all more or less cytotoxic and may impair the biocompatibility of chitosan [16]. In this regard, genipin extracted from gardenia fruits is obviously advantageous. As a water-soluble bi-functional crosslinker, it reacts promptly with chitosan. The resulting crosslinked products have better biocompatibility and stability for use in biomedical applications [7,16].

Another approach to obtain chitosan materials with enhanced chemical stability is to use the blend of chitosan with other polymers [6]. For instance, chitosan–sodium alginate nanofiber membranes prepared by a freeze-drying method and crosslinked by glutaraldehyde exhibited a decreased degree of swelling in various pH solutions with increasing amount of sodium alginate in the blend [17]. Similarly to the binary blends, a ternary composition of chitosan/montmorillonite grafted with lactic acid showed good stability regardless of pH of the medium [18], and reduced enzymatic biodegradation [19], indicating the potential uses of the novel hybrid in controlled drug delivery and tissue engineering applications.

In the field of textile industry, chitosan fiber, regardless of its use as a pure product or blends with other staple fibers, has the poor resistance to the treatment under acidic conditions and peroxide bleaching [8,20]. Acidic treatment and oxidative bleaching can lead to the weight loss of chitosan fiber and the reduced content of chitosan fiber in blended textiles, which exert negative effects on the functional and mechanical properties of the blends. Therefore, to improve the resistance of chitosan fiber to acid and hydrogen peroxide has become an urgent task. In previous reports [21,22,23,24], glyoxal, glutaraldehyde and epichlorohydrin had been used to crosslink chitosan fiber in the post-treatment stage after wet spinning. After crosslinking treatment, chitosan fiber showed an enhanced acid resistance proved by its reduced swelling in aqueous acetic acid. Our recent work showed that the solubility of chitosan fiber in sulfuric acid solution was remarkably decreased by means of crosslinking with a water-soluble diepoxy compound [8].

As chitosan fiber is applied to develop medical and healthy clothing as well as bioactive dressings, its good antibacterial activity is a great advantage. However, its antioxidant activity needs to be upgraded. Antioxidant activity is one of the most important properties of bioactive textiles. The textiles containing antioxidant compounds can act as a reservoir system capable of progressively deliver the active substances to the skin layers during the process of contact with human skin [25]. These active substances can scavenge free radicals from skin degeneration and present in atmosphere, and thus protect the skin from oxidative stress, inflammation and aging [26]. The textiles possessing antioxidant function can be used for preparing clothes for people with allergies and other skin diseases. Chitosan polymer is a good carrier for loading antioxidant compounds. When chitosan polymer with high molecular weight is used to manufacture a pure chitosan fiber, or mixed with viscose to prepare a chitosan-containing bicomponent fiber, the resulting fibers have poor scavenging activity [27,28]. It has been proved that to incorporate the active ingredients (e.g., polyphenolic compounds) of natural plant extracts onto the polysaccharide backbone of chitosan can enhance the antioxidant activity of chitosan [28,29,30,31,32,33]. In our earlier study, lac dye whose major active constituents are hydroxyl-containing anthraquinone compounds was successfully used to endow chitosan fiber with the good antioxidant activities determined by the DPPH radical scavenging assay (DPPH, 2,2-diphenyl-1-picrylhydrazyl) and the ABTS radical decolorization assay (ABTS, 2,2′-azino-bis [3-ethylbenzothiazoline-6-sulphonic acid] diammonium salt) [27].

In order to address the drawbacks present in the development of chitosan fiber textiles, this study aims to use a water-soluble aziridine crosslinker to indroduce the crosslinking between chitosan molecules for the enhenced acid resistance of chitosan fiber, and to employ lac dyeing to improve the bioactivities of the crosslinked chitosan fiber. In more concrete terms, the main application conditions and crosslinking mechanism of the aziridine crosslinker were discussed, and the weight loss of the crosslinked chitosan fiber in acidic solution was used to characterize its acid resistance. Additionally, the adsorption isotherm, adsorption mechanism and building-up property of lac dye on the crosslinked chitosan fiber were studied. Finally, the effects of crosslinking and dyeing on the antibacterial and antioxidant activities of chitosan fiber were determined.

## 2. Materials and Methods

### 2.1. Materials

The 2.22 dtex × 38 mm staple chitosan fiber was bought from Shandong Weifang Youngchito Bio. Co. Ltd., Weifang, China. Prior to chemical crosslinking, fiber samples were treated in the solution containing 0.5 g/L sodium bicarbonate and 0.5 g/L Leveling Agent O (polyoxyethylene alkyl ether) at 80 °C for 40 min using a 50:1 liquor ratio (the ratio of liquor volume to fiber weight) with the aim of removing the spin oils added to the fiber during the post-spinning process. After this treatment, fibers samples were thoroughly rinsed in tap water, and allowed to dry in the open air.

**Figure 1:**
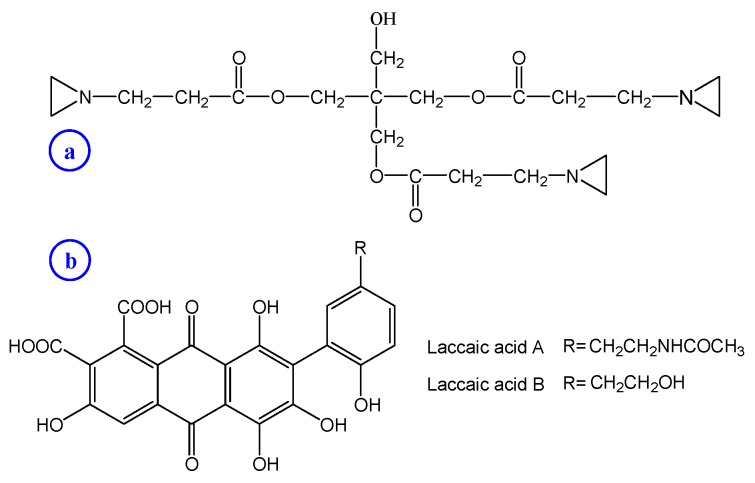
Chemical structures of Crosslinker SaC-100 (**a**) and two major colorant species present in lac dye (**b**).

A commercial water-soluble aziridine crosslinker (Crosslinker SaC-100, pentaerythritol tris[3-aziridinopropionate]) with a purity of 99% was provided by Shanghai UN Chemical Co. Ltd., Shanghai, China. The chemical structure of Crosslinker SaC-100 is shown in Figure 1a. Lac dye was purchased from Yunnan Tonghai Young’s Natural Products Co. Ltd., Tonghai, China and used as received. The chemical structures of two main anthraquinone-based ingredients (laccaic acids A and B) in lac dye are shown in Figure 1b. Leveling Agent O was provided by Jiangsu Haian Petrochemical Factory, Haian, China. 2,2′-Azino-bis (3-ethylbenzothiazoline-6-sulphonic acid) diammonium salt (ABTS) was obtained from Shanghai D and B Chemicals Technology Co. Ltd., Shanghai, China. Potassium persulfate sodium sulfate sulfuric acid, citric acid, potassium dihydrogen phosphate, sodium hydrogen phosphate, disodium hydrogen phosphate and sodium bicarbonate were of analytical reagent grade. Nutrient agar and nutrient broth were obtained from Sinopharm Chemical Reagent Co. Ltd., Shanghai, China and Shanghai Sincere Biotech Co. Ltd., Shanghai, China, respectively.

### 2.2. Crosslinking and Dyeing

Both the crosslinking of chitosan fiber and the adsorption and dyeing of lac dye were carried out in the sealed and conical flasks immersed in the XW–ZDR low-noise oscillated dyeing machine (Jiangsu Jingjiang Xingwang Dyeing and Finishing Machinery Factory, Jiangjiang, China). The liquor ratio was 100:1. At the end of crosslinking, adsorption and dyeing, the treated chitosan fibers were fully washed in distilled water and then allowed to dry in the open air.

#### 2.2.1. Crosslinking Treatment

To evaluate the temperature dependence of the crosslinking of chitosan fiber, crosslinking treatment was carried out at five temperatures (30, 40, 50, 60 and 70 °C) for 60 min using 8 g/L Crosslinker SaC-100. To assess the effect of duration on the crosslinking of chitosan fiber, crosslinking treatment was conducted at 60 °C for different times (20, 40, 60, 80, 100 and 120 min) using 8 g/L Crosslinker SaC-100. To study the effect of the dosage of Crosslinker SaC-100 on the crosslinking of chitosan fiber, crosslinking treatment was made at 60 °C for 60 min using six Crosslinker SaC-100 dosages (0, 2, 4, 6, 8 and 10 g/L).

In addition, the sample crosslinked with 8 g/L Crosslinker SaC-100 at 60 °C for 60 min was employed for the analysis of Fourier transform infrared (FT-IR), the experiments of the adsorption and dyeing of lac dye, and the bioactivity tests.

#### 2.2.2. Adsorption and Dyeing Experiments

The equilibrium adsorption isotherms for lac dye on the uncrosslinked and crosslinked chitosan fibers were measured in a series of lac dye solutions of various concentrations (2%–40% owf [on the weight of fiber]) at 50 °C in the presence of 5 g/L sodium sulfate. The pH was adjusted to 5.33 by citric acid-disodium hydrogen phosphate buffer. The isotherms were determined on the basis of the adsorption for 120 min as the previous tests showed that the equilibrium adsorption was reached in 60 min.

In view of practical application conditions, the building-up properties of lac dye on the chitosan fibers with and without crosslinking were determined in a temperature-rise process in place of a constant temperature process. The building-up properties were measured in the dye solutions of the concentration of 2%–40% owf at pH 5.33. The dyeing started at 30 °C, the temperature was raised to 50 °C at a rate of 1 °C/min, and at this temperature the dyeing continued for 60 min.

### 2.3. Measurements

#### 2.3.1. Acid Resistance Test

The acid resistance of the crosslinked chitosan fiber was characterized by its weight loss in acidic solution. The sample was treated in 0.1 mol/L sulfuric acid solution at 80 °C for 60 min using a liquor ratio of 100:1. After this treatment, the fiber residue was obtained by filtering the solution. During the process of filtering, the fiber residue was completely washed with distilled water until the filtered water became neutral. Afterwards, the original fiber and the fiber residue obtained from acidic treatment were first dried in an oven at 60 °C for 40 min, and subsequently at 105 °C for 120 min. The weight loss of the crosslinked chitosan fiber during acidic treatment was calculated using Equation (1).

(1) $$\text{Weight loss }(\%)=100\times \frac{W_{0}-W_{1}}{W_{0}}$$

where *W*_0_ and *W*_1_ are the dry weights of the original fiber and fiber residue, respectively.

#### 2.3.2. FT-IR Analysis

The Fourier transform infrared (FT-IR) spectra of chitosan fiber powders were recorded by the Nicolet 5700 FT-IR spectrometer (Thermo Fisher Scientific Inc., Waltham, MA, USA) from 4000 to 400 cm^−1^ using potassium bromide pellets.

#### 2.3.3. Adsorption Measurements

The absorbance at the maximum absorption wavelength of lac dye solutions was measured using the Shimadzu UV-1800 UV–Vis spectrophotometer (Shimadzu Co., Kyoto, Japan). Using a previously established absorbance and concentration relationship of lac dye solutions, the quantity of lac dye in solution was able to be calculated, and the percentage of exhaustion was determined using Equation (2), where m_0_ and m_1_ are the quantities of lac dye before and after adsorption. The quantity of lac dye on chitosan fiber was calculated by the difference in the initial and final concentrations of lac dye in solution as well as the weight of the dried fiber.

(2) $$\text{Exhaustion }(\%)=100\times \frac{m_{0}-m_{1}}{m_{0}}$$

#### 2.3.4. Bioactivity Tests

The samples obtained in the experiments of building-up properties were used to evaluate of antioxidant and antibacterial activities.

The antioxidant activity was determined by the ABTS radical decolorization assay according to a previously reported method [27]. ABTS (7 mM) was dissolved in water. The ABTS radical cation (ABTS˙^+^) was produced by reacting ABTS stock solution with 2.45 mM potassium persulfate. The mixture was stored in the dark at room temperature for 12–16 h prior to use. Before usage, the ABTS˙^+^ solution was diluted with a phosphate buffer (0.1 M, pH 7.4) to reach an absorbance of 0.700 ± 0.025 at 734 nm. Subsequently, 10 mg of the fiber sample was added to 10 mL of ABTS˙^+^ solution. After 30 min, the absorbance was measured at 734 nm. Lower absorbance of the reaction mixture indicates higher ABTS˙^+^ scavenging activity. The scavenging capability of ABTS˙^+^ was calculated using Equation (3).

(3) $$\text{Antioxidant activity }(\%)=\frac{A_{\text{ctrl}}-A_{\text{spl}}}{A_{\text{ctrl}}}\times 100$$

where *A*_ctrl_ is the initial absorbance of the ABTS˙^+^, and *A*_spl_ is the absorbance of the remaining ABTS˙^+^ in the presence of the fiber sample.

The antibacterial activities of chitosan fiber samples were evaluated according to GB/T 20944.3-2008. *Staphylococcus aureus* (*S. aureus*) and *Escherichia coli* (*E. coli*) are the common pathogenic bacteria in daily life. The former is a gram-positive coccal bacterium, and it can cause a range of illnesses from minor skin infections. The latter is a gram-negative, facultative anaerobic. Although most *E. coli* strains do not cause disease, virulent strains can cause illness through contact infections. Therefore, the two pathogenic strains were adopted in this test. The measurements are briefly described as follows:

The specimens of fiber fragments were soaked in the conical flasks with bacteria oscillating in a shaker at a desired temperature (*E. coli* at 30 °C and *S. aureus* at 24 °C) for 24 h. Afterwards, the bacteria solutions were diluted 1000 times and inoculated onto the agar plates which were stored for a desired time (*E. coli* at 24 h and *S. aureus* at 48 h) at 37 °C. Finally, the quantity of the visually bacterial colonies was counted, and the antibacterial activity was calculated using Equation (4).

(4) $$\text{Antibacterial activity }(\%)=\frac{N_{\text{ctrl}}-N_{\text{spl}}}{N_{\text{ctrl}}}\times 100$$

where *N*_ctrl_ and *N*_spl_ are the quantities of the visually bacterial colonies of standard cotton fabric and tested sample, respectively.

## 3. Results and Discussion

### 3.1. Crosslinking of Chitosan Fiber

#### 3.1.1. Crosslinking Conditions and Acid Resistance of Chitosan Fiber

Three important crosslinking conditions (temperature, duration and crosslinker dosage) employed for the crosslinking treatment of chitosan fiber with the aziridine crosslinker, Crosslinker SaC-100 were discussed. The weight loss of the crosslinked chitosan fiber in acidic solution was used to estimate the extent of crosslinking, and characterize the acid resistance of chitosan fiber.

**Figure 2:**
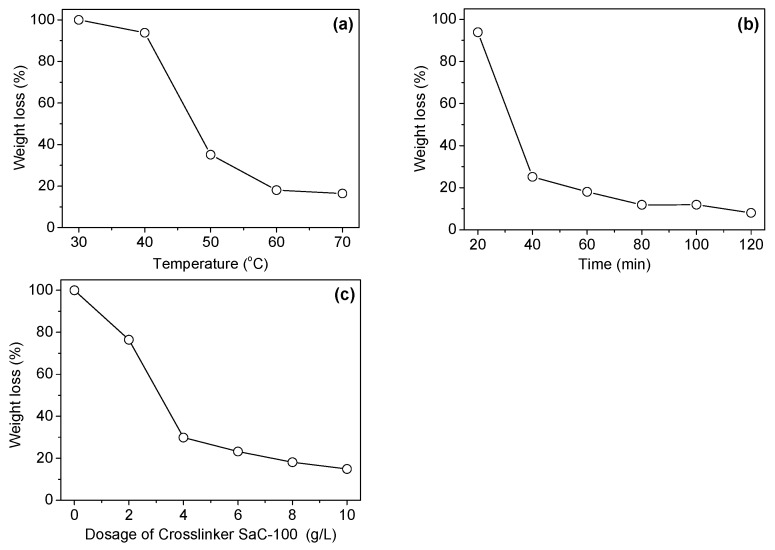
Effects of temperature (**a**), time (**b**) and Crosslinker SaC-100 dosage (**c**) used in crosslinking treatment on the weight loss of the crosslinked chitosan fiber in acidic solution.

Figure 2a shows the dependence of the weight loss of chitosan fiber on the application temperature of the aziridine crosslinker. At low temperatures (30 and 40 °C), the aziridine crosslinker was not able to introduce an efficient crosslinking between the molecules of chitosan fiber. With rising application temperature of the crosslinker, the extent of crosslinking was significantly enhanced, and accordingly the weight loss of the crosslinked chitosan fiber in acidic solution decreased remarkably. The crosslinking treatment at 60 or 70 °C was able to impart good acid resistance to chitosan fiber. The low crosslinking extent of the aziridine crosslinker at low temperatures is preliminarily considered as a consequence of its high molecular weight (427). Our previous work showed that the water-soluble diepoxy crosslinker (Crosslinker EH) with a molecular weight of 228 had relatively good crosslinking effect [8]. Thus, it is reasonable to assume that the adequate swelling of chitosan fiber induced by increasing temperature is essential for the diffusion of the aziridine crosslinker into fiber interior, and the formation of the crosslinking between the molecules of chitosan fiber.

Figure 2b shows the effect of crosslinking treatment time on the weight loss of the crosslinked chitosan fiber in acidic solution. Clearly, the treatment for a short time (20 min) was not able to provide an efficient crosslinking. After the treatment for 40 min, the weight loss of chitosan fiber decreased gradually with increasing reaction time. However, with a further increase in the time ranging from 80 to 120 min, the weight loss did not show an obvious decrease, indicating that the reaction between chitosan fiber and Crosslinker SaC-100 tends to be complete. The above phenomena reveal that enough time is necessary for the crosslinker to be adsorbed by chitosan fiber, diffuse into chitosan fiber, and construct the conjugation between chitosan molecules.

Figure 2c shows the effect of crosslinker dosage on the stability of the crosslinked chitosan fiber in acidic solution. As shown in Figure 2c, the stability of the treated fiber to acid was greatly affected by the dosage of Crosslinker SaC-100. The weight loss of chitosan fiber in acidic solution decreased gradually with increasing dosage of Crosslinker SaC-100. When the dosage of Crosslinker SaC-100 reached 8 and 10 g/L, the weight loss of the treated fiber decreased to 18.1% and 14.9%, respectively. These dosages imparted good acid resistance to chitosan fiber.

Based on the above research results, the crosslinking treatment should be carried out at 60 °C for 60–80 min, and the crosslinking extent of chitosan fiber may be controlled by the crosslinker dosage according to the desired requirements.

#### 3.1.2. Crosslinking Mechanism

**Figure 3:**
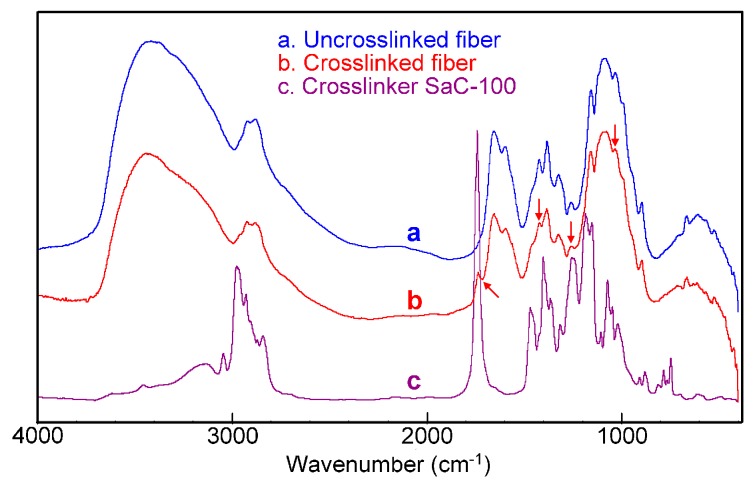
Fourier transform infrared (FT-IR) spectra of the uncrosslinked chitosan fiber (**a**), the crosslinked chitosan fiber (**b**) and Crosslinker SaC-100 (**c**).

The FT-IR analysis can provide the information about the reaction of Crosslinker SaC-100 with chitosan fiber. Figure 3 demonstrates the FT-IR spectra of the uncrosslinked chitosan fiber, the chitosan fiber crosslinked with 8 g/L Crosslinker SaC-100, and Crosslinker SaC-100. The important absorption bands of the untreated chitosan fiber (spectrum a) were observed as follows: 3415 cm^−1^ (overlapping bands of O–H and N–H stretching), 1654 cm^−1^ (amide I, C=O stretching), 1600 cm^−1^ (amide II, N–H bending), 1425 cm^−1^ (–NH deformation vibration in –NH_2_), 1323 cm^−1^ (amide III, C–N stretching), 1260 cm^−1^ (–OH deformation), 1156 and 895 cm^−1^ (bridge C–O–C), and 1082 and 1031 cm^−1^ (C–O bonds of CH–OH and C–OH) [8,11,34,35,36,37,38].

Compared with the uncrosslinked fiber, the crosslinked sample (spectrum b) displayed an obvious shift of the O–H stretching band from 3415 to 3441 cm^−1^, and a clear variation in the absorption intensity of the bands at 1260, 1082 and 1031 cm^−1^, implying that the hydroxyl groups in chitosan react with the aziridine crosslinker [8,11]. Additionally, after crosslinking, chitosan fiber displayed a slightly decreased absorption intensity of the –NH deformation vibration in –NH_2_ at 1425 cm^−1^ [36], indicating that a small number of the amino groups in chitosan participate in crosslinking reaction. It is worth noting that a novel band at 1739 cm^−1^ (spectrum b) was found for the crosslinked fiber, which should be ascribed to the ester groups in the aziridine crosslinker (spectrum c). From the above discussions, it can be concluded that the reaction of the aziridine crosslinker with the hydroxyl groups in chitosan predominantly contributes to the formation of crosslinking between chitosan molecules.

### 3.2. Lac Dyeing of Chitosan Fiber

#### 3.2.1. Equilibrium Adsorption Isotherms of Lac Dye

The research on the equilibrium adsorption isotherms of lac dye on chitosan fiber helps to understand its adsorption mechanism and interactions with chitosan fiber, and to control its process. Although little research has been done on the equilibrium adsorption of dyes on chitosan fiber, large quantities of investigations on the adsorption of anionic dyes on chitosan polymers have been reported [39,40,41,42,43]. Most of these researches pointed out that the electrostatic interactions between the anionic groups in dyes and the positively charged amino groups in chitosan chains had great contribution to dye adsorption, which were proved by the Langmuir [39,40,41,42] and Redlich–Peterson [43] isotherms.

**Figure 4:**
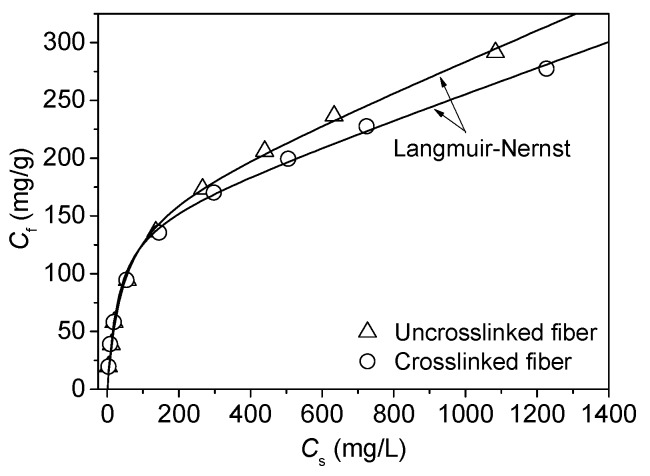
Adsorption isotherms of lac dye on the uncrosslinked and crosslinked chitosan fibers.

Lac dye is anionic because of the presence of carboxyl groups in its structure. In our previous report, the Langmuir–Nernst equation (the dual adsorption equation consisting of Langmuir and partition models) was found to be the most appropriate model to describe the behaviors of the adsorption of lac dye on chitosan fiber in the case that pH was not adjusted [27]. In this work, the pH of lac dye solution was adjusted to 5.33 by a buffer. The adsorption isotherms of lac dye on the uncrosslinked and crosslinked chitosan fibers are depicted in Figure 4. In order to analyze these isotherms well, three isothermal models, namely Langmuir, Freundlich, and Langmuir–Nernst, were used to fit these adsorption data.

The Langmuir isotherm is expressed as:

(5) $$C_{f}=\frac{SK_{L}C_{s}}{1+K_{L}C_{s}}$$

where *C*_f_ (mg/g) and *C*_s_ (mg/L) are the concentrations of lac dye on chitosan fiber and in solution, respectively; *S* is the saturation concentration of lac dye on chitosan fiber; *K*_L_ is the Langmuir affinity constant.

The empirical Freundlich isotherm is:

(6) $$C_{f}=K_{F}C_{s}{}^{n}$$

where *K*_F_ is the Freundlich affinity constant, and *n* is an indicator of adsorption intensity or surface heterogeneity.

The Langmuir–Nernst dual model is described by the following expression:

(7) $$C_{f}=C_{P}+C_{L}=K_{P}C_{S}+\frac{SK_{L}C_{s}}{1+K_{L}C_{s}}$$

where *C*_P_ and *C*_L_ are the concentrations of lac dye on chitosan fiber by Nernst and Langmuir adsorption, respectively; *S* is the saturation concentration of lac dye on chitosan fiber by Langmuir adsorption; *K*_P_ and *K*_L_ are the partition coefficient and the Langmuir affinity constant, respectively.

The nonlinear least-squares fitting procedure was used to fit the experimental adsorption data in Figure 4 by three adsorption models. To evaluate the fitting results, the normalized deviations (*ND*) of the experimental values used to assess the extent of fitting were calculated according to Equation (8).

(8) $$ND\;(\%)=100\times \frac{1}{N}\sum_{i=1}^{N}\left(\frac{\left|C_{f,\exp,i}-C_{f,calc,i}\right|}{C_{f,\exp,i}}\right)$$

where *C*_f,exp,i_ and *C*_f,calc,i_ are the experimental and calculated values (the quantity of lac dye adsorbed by chitosan fiber), respectively; the index “*i*” refers to the sequence number of adsorption data; *N* is the total number of data sets.

Table 1 shows that the Langmuir–Nernst equation had the lowest normalized deviation. In Figure 4, the Langmuir–Nernst curve almost went through all the experimental data exactly. The results indicate that the Langmuir–Nernst isotherm is the most appropriate model to describe the adsorption behavior of lac dye on the uncrosslinked and crosslinked chitosan fibers. According to this model, it can be concluded that the electrostatic interactions between lac dye and chitosan fiber contribute to Langmuir adsorption, whereas the non-electrostatic interactions between lac dye and chitosan fiber contribute to Nernst partition adsorption.

The p*K*_a_ values of the primary amine groups in chitosan and the carboxyl groups in lac dye are approximately 6.3 [44,45] and 5.96 [46], respectively. At these two pHs corresponding to the p*K*_a_ values, the ionized fractions of primary amine groups and carboxyl groups are 50%. In the present study, the pH of lac dye solution is 5.33, close to the p*K*_a_ values of the two groups. Therefore, the carboxyl groups in lac dye can combine with the protonated amino groups in chitosan fiber by virtue of ion-ion interactions, contributing to Langmuir adsorption of lac dye. In addition, chitosan fiber contains a large number of amino and hydroxyl groups, and lac dye has multiple hydrogen groups. These make it possible that lac dye interacts with chitosan fiber by virtue of hydrogen bonding. Hydrogen bonding as well as van der Waals forces can contribute to Nernst partition adsorption of lac dye.

**Figure 5:**
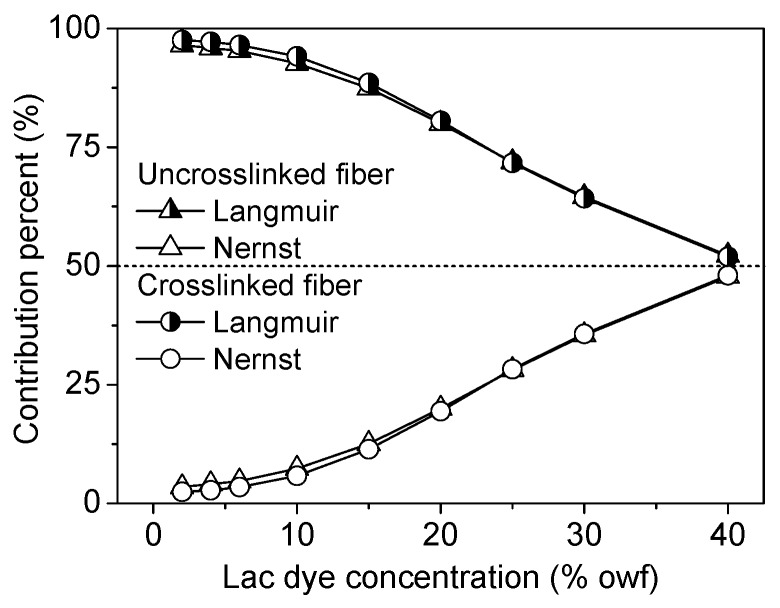
Contribution of Langmuir and Nernst adsorption to lac dye adsorption on the uncrosslinked and crosslinked chitosan fibers.

According to our previously reported method [27,47], the percent of the contribution of Langmuir or Nernst adsorption to total adsorption was calculated. Figure 5 shows that the contribution of Langmuir adsorption to total adsorption decreased with increasing initial lac dye concentration, whereas that of Nernst adsorption increased. The contribution percent of Langmuir adsorption was higher than the value that we reported earlier [27] as a lower pH was used in the present study. The comparison of the contribution of Langmuir adsorption between the uncrosslinked and crosslinked chitosan fibers showed no difference, revealing that the crosslinking of chitosan fiber with the aziridine crosslinker does not change the nature of lac dye adsorption.

Table 2 shows the adsorption parameters for the Langmuir–Nernst model. As can be seen in Table 2, the crosslinking with the aziridine crosslinker slightly decreased the Langmuir adsorption saturation of lac dye. This can be explained by the fact that a small number of the amino groups in chitosan participate in the crosslinking reaction as mentioned above. A slight reduction in the saturation of Langmuir adsorption gives rise to the minute variation of *K*_L_ and *K*_P_ values.

#### 3.2.2. Building-up Properties of Lac Dye

**Figure 6:**
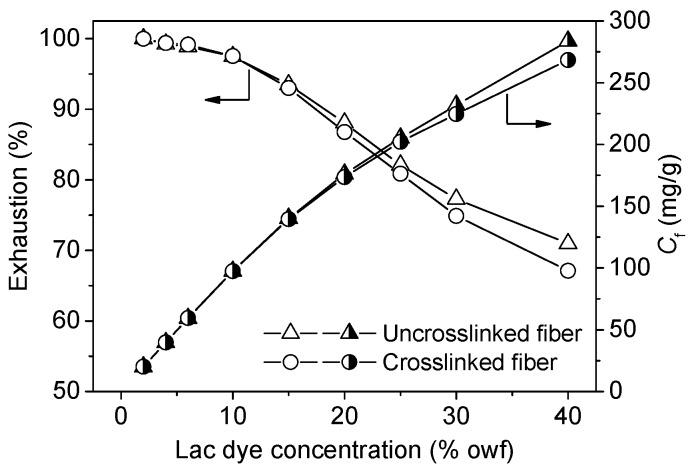
Building-up properties of lac dye for the uncrosslinked and crosslinked chitosan fibers.

The functional dyes with good building-up performance are helpful in imparting dark shades and good functionalities to chitosan fiber at their low dosages and processing cost. Figure 6 shows that the quantity of the adsorption of lac dye on both the uncrosslinked and crosslinked chitosan fibers continued to increase with increasing lac dye concentration. This indicates that lac dye has good building-up performance on chitosan fibers. Figure 6 also shows that lac dye still had a high exhaustion even at its high dosage. These observations imply that lac dye exhibits very high adsorption capability and utilization rate when applied to the uncrosslinked and crosslinked chitosan fibers.

As shown in Figure 6, the quantity of lac dye adsorption had was no difference between the uncrosslinked and crosslinked chitosan fibers when the concentration of lac dye was less than or equal to 15% owf. However, lac dye displayed slightly lower exhaustion and adsorption quantity for the crosslinked fiber than the uncrosslinked fiber as its dosage exceeded 20% owf. This reveals that the slightly reduced content of the amino groups in the crosslinked chitosan fiber causes a minimal decrease in the adsorption quantity of lac dye when a considerable number of dyes surround the fiber aggregates. Nevertheless, this may not affect the application of lac dye for the functional dyeing of the crosslinked chitosan fiber because the dye dosage exceeding 20% owf is a rare case in practical production.

### 3.3. Bioactivities of Chitosan Fiber

**Figure 7:**
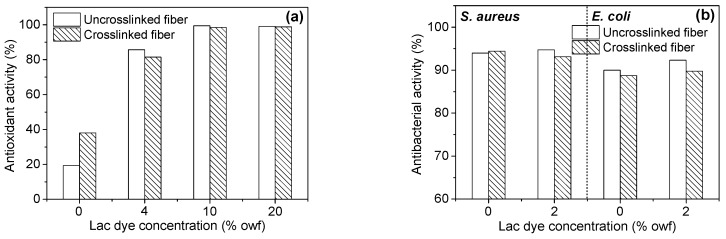
Antioxidant (**a**) and antibacterial (**b**) activities of the crosslinked and dyed chitosan fibers.

Antioxidant and antibacterial properties are two important functionalities of bioactive textile fibers. Pure chitosan fiber with high molecular weight has very poor free radical scavenging activity [11], which is a great weakness for its functional applications. The poor antioxidant activity of the untreated chitosan fiber was validated again by the results of Figure 7a. The crosslinking with the aziridine crosslinker endowed chitosan fiber with improved antioxidant activity. After lac dyeing, the antioxidant activity of the uncrosslinked and crosslinked fibers was greatly enhanced. Moreover, antioxidant activity increased with increasing dosage of lac dye until the maximum value was reached at a lac dye dosage of 10% owf. Lac dye consists of the anthraquinone compounds containing multiple phenolic hydroxyl groups. The hydroxyl-containing anthraquinone compounds can exhibit antioxidant activity due to the presence of hydrogen-donating phenolic hydroxyl groups in their parent structures [48].

Figure 7b shows the antibacterial activities of the uncrosslinked and crosslinked chitosan fibers against *S. aureus* and *E. coli*. From Figure 7b, it is evident that chitosan fiber exhibited good antibacterial activities irrespective of crosslinking and dyeing, its antibacterial activity against *S. aureus* was better than that against *E. coli*, and the minimum antibacterial activities against *S. aureus* and *E. coli* were 93.1% and 88.7%, respectively. Compared with the uncrosslinked samples, the crosslinked samples showed a very low rate of variation in antibacterial activity from −2.78% to 0.46%. In addition, the variation rate of the antibacterial activity between the undyed and dyed samples was also very small and in the range of −1.36% to 2.57%. Thus, it is considered that crosslinking and lac dyeing have no impart on the good antibacterial activity of chitosan fiber.

## 4. Conclusions

The chitosan fiber possessing enhanced acid resistance and good bioactivities was successfully prepared by the crosslinking with a water-soluble aziridine crosslinker followed by the dyeing with natural lac dye consisting of polyphenolic anthraquinone compounds. The crosslinking treatment should be carried out at 60 °C for 60–80 min, and the crosslinking extent of chitosan fiber was able to be controlled by the crosslinker dosage. The crosslinked fiber was able to be dyed under the weakly acidic condition, which provided high dye adsorption quantity and dye utilization rate. The equilibrium adsorption analysis showed that the Langmuir–Nernst isotherm was the best model to describe the adsorption behavior of lac dye, and Langmuir adsorption had greater contribution to total adsorption than Nernst adsorption. Lac dyeing endowed chitosan fiber with good antioxidant activity, and had no impact on the good inherent antibacterial activity of chitosan fiber. This study points out that crosslinking and lac dyeing enable chitosan fiber to be applicable for the development of bioactive, healthy and hygienic chitosan fiber textiles.

## Figures and Tables

**Table 1:** Normalized deviation of three isotherm models.

Model	*ND* (%)	
	Uncrosslinked	Crosslinked
Langmuir	22.65	24.43
Freundlich	10.21	11.01
Langmuir + Nernst	6.60	6.12

**Table 2:** Parameters in the Langmuir–Nernst equation for the adsorption of lac dye on chitosan fibers.

Fiber	*K*_L_ (L/mg)	*S* (mg/g)	*K*_P_ (L/mg)
Uncrosslinked	0.0245	159.7	0.130
Crosslinked	0.0327	149.6	0.110
